# Kefiran as a Multifunctional Biopolymer: Green Extraction, Structural Characterization and Application in Phenolic-Loaded Complex Coacervates

**DOI:** 10.3390/foods15122138

**Published:** 2026-06-13

**Authors:** Paul K. Agyei, Yemane H. Gebremeskal, Anastasia A. Mentova, Tatyana F. Chernykh, Tarek N. Soliman, Hassan Barakat, Khalid A. Alsaleem, Tamer M. El-Messery, Mohamed S. Boulkrane

**Affiliations:** 1 Faculty of Biotechnologies, ITMO University, Saint Petersburg 191002, Russia; paulagyei27@gmail.com (P.K.A.); yhgebremeskal@itmo.ru (Y.H.G.); 2 Food Science and Technology Department, Hamel Malo Agricultural College, Keren, Eritrea; 3 Department of Microbiology, Saint-Petersburg State Chemical and Pharmaceutical University, Saint Petersburg 197376, Russia; hertz7581@gmail.com (A.A.M.); tatiana.odegova@pharminnotech.com (T.F.C.); 4 Dairy Department, Food Industries and Nutrition Research Institute, National Research Center, Cairo 12622, Egypt; tariknour.nrc@gmail.com; 5 Department of Food Science and Human Nutrition, College of Agriculture and Food, Qassim University, Buraydah 51452, Saudi Arabia; haa.mohamed@qu.edu.sa (H.B.); k.alsaleem@qu.edu.sa (K.A.A.)

**Keywords:** Kefiran, complex coacervation, ultrasound-assisted extraction, antioxidant activity, antimicrobial activity, exopolysaccharide, food supply

## Abstract

This study examined Kefiran, an exopolysaccharide derived from milk kefir grains, as a novel biopolymer for encapsulating phenolic extracts from sunflower cake and its antimicrobial properties in the development of natural and functional food ingredients. Kefiran was obtained from kefir grains using three extraction protocols: hot water (M1), hot water with 30% trichloroacetic acid (M2), and mild heat combined with ultrasound at 60 °C (M3). The ultrasound-assisted method produced the highest carbohydrate concentration. Spectrophotometric assays (phenol–sulfuric and Bradford), Fourier transform infrared spectroscopy, scanning electron microscopy, thermogravimetric analysis, and water-holding capacity were employed to characterize the composition, structure, and morphology of the extracts, revealing well-preserved polysaccharide fingerprints and a highly porous microstructure, consistent with their potential application in food systems. Kefiran was then evaluated as an encapsulating agent in complex coacervation at pH 3.75, using three Kefiran-based wall formulations (M1, M2, and M3) with gum arabic and whey protein isolate (WPI) as co-wall materials, and their performance was compared with gum arabic and WPI controls. Across formulations, coacervate microcapsules achieved high encapsulation efficiencies (83–93%), tunable particle sizes, and predominantly negative zeta potentials, indicative of good colloidal stability. The Kefiran extract and coacervate microcapsules demonstrated significant antioxidant and antimicrobial activity against *Staphylococcus aureus*, *Escherichia coli*, *Pseudomonas aeruginosa*, and *Candida albicans*, with minimum inhibitory concentrations ranging from 250 to 1000 µg/mL. The findings support ultrasound-extracted Kefiran as a multifunctional biopolymer suitable for bioactive delivery and as a natural antimicrobial component in advanced functional food formulations.

## 1. Introduction

Exopolysaccharides (EPSs) are high-molecular-weight biopolymers synthesized and secreted by diverse microorganisms, comprising approximately 40–95% of the polymeric substances secreted into the surrounding environment [1]. These long-chain polysaccharides consist of branched, repeating units of sugars and sugar derivatives, primarily glucose, galactose, and rhamnose, in varying proportions [2]. EPSs can be classified as either homopolysaccharides or heteropolysaccharides based on monosaccharide composition [3,4]. For instance, dextran and bacterial cellulose are homopolysaccharides containing D-glucose linked by α-(1→6) bonds and α-(1→4) bonds, respectively. These linkages are repeated in dextran and bacterial cellulose and can also have different branches [5,6]. Owing to their structural diversity and functionality, EPSs have found broad industrial applications, such as stabilizers and drug delivery systems [7]. In microbial environments, EPSs serve structural and defensive functions and are commonly extracted for use as edible films, drug delivery vehicles, and emulsifiers, among other applications. Their wide applications stem from their various functional properties, such as biocompatibility, biodegradability, and non-toxicity, and they often exhibit inherent biological activities, including immunomodulatory, prebiotic, and antioxidant properties [8,9]. Because of their adaptability, they are excellent choices for use in the food, pharmaceutical, nutraceutical, and biomedical industries [10]. Research on microbial polysaccharides as renewable substitutes for synthetic polymers has expanded in recent years due to consumer desire for clean-label, natural, and sustainable functional materials [11]. To fully maximize the industrial potential of EPSS without compromising environmental sustainability, green extraction methodologies, improved structural characterization, and multifunctional applications are necessary. Extraction processes significantly influence the yield and physical properties of Kefiran, but not its chemical structure. Conventional hot-water extraction and novel ultrasound-assisted extraction procedures have distinct advantages and limitations regarding environmental sustainability, extraction efficiency, and structural preservation. Green extraction approaches, such as ultrasound-assisted mild thermal processing, are recommended for their reduced solvent consumption, lower energy requirements, shorter extraction time, and higher retention of bioactive properties. Assessing these approaches is essential to establishing scalable, sustainable industrial production strategies for Kefiran. Consequently, increasing interest has been directed toward identifying and developing novel EPSS for industrial applications.

Among the various types of exopolysaccharides, Kefiran, produced by *Lactobacillus kefiranofaciens* and related species during the fermentation of kefir grains, has attracted significant interest due to its structure and biological properties [12,13]. Kefiran is composed of approximately equal amounts of glucose and galactose units linked by (1,3), (1,4), and (1,6) glycosidic bonds [14]. Structural analyses using nuclear magnetic resonance (NMR), Fourier Transform Infrared Spectroscopy (FTIR), and X-ray diffraction have confirmed the presence of carboxyl, hydroxyl, and other functional groups that contribute to Kefiran’s unique physicochemical and biological properties [15]. These structural characteristics are critical in determining its rheological behavior, film-forming ability, encapsulation potential, and interaction with proteins or phenolic compounds. Several studies have highlighted Kefiran’s antioxidant, anticancer, antimicrobial, and immunoregulatory properties, coupled with its biopolymer nature as a functional carrier in food and biomedical formulations [16,17]. The antimicrobial activity of Kefiran and its derivative formulations helps address the challenge of antimicrobial resistance (AMR) by providing non-toxic, natural inhibitory effects against harmful microorganisms [18,19]. Kefiran has been reported to inhibit the growth of pathogenic Gram-positive bacteria (e.g., *S. aureus*) and Gram-negative bacteria (e.g., *E. coli*), as well as fungi [20]. Antimicrobial activity has been attributed to its ability to inhibit the formation of microbial biofilms by disrupting microbial cell membranes [21].

Beyond its physicochemical properties, Kefiran’s multifunctionality as both a bioactive compound and biomaterial positions it as a unique material for advanced pharmaceutical and food delivery systems. Encapsulation technologies play an important role in protecting volatile bioactive compounds from environmental degradation and in improving their stability, bioavailability, and controlled release, ensuring that they exert their functions at specific sites and conditions while maximizing their efficacy [22]. Complex coacervation is a widely used technique in the food industry for encapsulating bioactive compounds (e.g., phenolic compounds, vitamins, and essential oils) and protecting them from degradation under specific environmental conditions. This technique involves the formation of complex coacervate microcapsules via electrostatic interactions between oppositely charged polymers (proteins and polysaccharides) [23]. The choice of encapsulation material, such as polysaccharides and proteins, and the encapsulation parameters, including pH and the ratio of wall material, are key in determining the encapsulation efficiency and the release kinetics of the encapsulate [24,25]. In parallel, the protein–polysaccharide complex coacervation system, particularly between whey protein isolate and gum arabic, has demonstrated efficient encapsulation performance due to favorable colloidal stability at acidic pH around 3.5–4.0 [26,27]. Integrating Kefiran into such systems could provide a dual benefit: enriching the wall material with a bioactive polysaccharide while leveraging electrostatic interactions with whey proteins to form protective matrices. Kefiran’s branched glucose–galactose structure enhances the formation of coacervates, yielding shells with improved porosity for sustained release and making it ideal for masking flavors, improving shelf life, or enabling site-specific delivery in functional foods and therapeutics [28]. Kefiran forms coacervate complexes with whey protein isolate (WPI) through electrostatic and hydrogen bonding, creating an osmotic gradient due to WPI’s positive charge and Kefiran’s neutrality, thereby forming microstructures that protect active compounds under acidic conditions [29]. Sunflower cake’s phenolic compounds have significant antioxidant potential; however, they are quite prone to degradation during processing and in the digestive system. Therefore, encapsulation within Kefiran-based complex coacervates may enhance the stability of phenolic compounds while increasing the functional uses of bioactive compounds derived from agricultural waste and Kefiran.

In light of this, the present study was designed with three primary objectives: (i) to compare hot-water, hot-water plus TCA, and ultrasound-assisted mild-heat protocols for Kefiran extraction in terms of yield, composition, structure, and morphology; (ii) to formulate Kefiran–whey protein–gum arabic complex coacervates for the encapsulation of sunflower cake phenolic extracts and to evaluate encapsulation efficiency, particle size, and surface charge; and (iii) to assess the antioxidant and antimicrobial activities of the resulting Kefiran and encapsulated systems against representative Gram-positive, Gram-negative, and fungal strains relevant to food safety. By linking extraction conditions, structural features, and biofunctional performance, the study provides a framework for using Kefiran as a multifunctional ingredient in phenolic delivery systems and clean-label antimicrobial formulations.

## 2. Materials and Methods

### 2.1. Chemicals and Reagents

All reagents and chemicals used in the study were of analytical grade and obtained from the Faculty of Biotechnologies laboratory, ITMO University (Saint Petersburg, Russia). These include 95% ethanol, distilled water, 98% sulfuric acid (H_2_SO_4_), glucose (C_6_H_12_O_6_), Coomassie brilliant blue reagent, bovine serum albumin (BSA), phenol (C_6_H_5_OH), gum arabic, and whey protein isolate, Folin–Ciocalteu phenol reagent, sodium carbonate, and n-hexane.

### 2.2. Sample Preparation and Defatting Sunflower Cake

The sunflower cake was ground into a fine powder in a blender. It was then subjected to defatting using the method described by Özcan et al. [30] with modifications. The fine-powdered sunflower cake was defatted using a Soxhlet extractor apparatus (LOIP, model 10 KSh 85/45, St. Peterburg, Russia). In total, 50 g of the sample was defatted using 250 mL of n-hexane, a highly volatile organic solvent, in a Soxhlet extractor apparatus. The defatting process was carried out at 69 °C until the solvent in the Soxhlet siphon became colorless. The extraction cycles ranged from 8 to 12. The extraction of solvent from the powdered sunflower was carried out using a rotary evaporator at a temperature of 40 °C. The n-hexane extract was recycled, and the defatting procedure was repeated. The point at which the reflux turns clear marks the maximum extraction time for each solvent. After extraction, the solvent was removed using a rotary evaporator under moderate vacuum. After that, the defatted sample was collected for subsequent extraction.

#### 2.2.1. Extraction of Phenolic Compounds from Sunflower Cake

The ultrasound-assisted extraction technique, with slight modifications based on the methods described by El Baakili et al. [31], was employed in this study using a Stegler Ultrasonic bath 3DT (3 L capacity, 120 W, 40 kHz, NV-LAB, Moscow, Russia). In a 1 L capped flat-bottomed flask, 50 g of defatted sunflower powder was mixed with 500 mL of distilled water and sonicated for 20 min. After extraction, the resulting mixture was centrifuged (Eppendorf 5810 R, Eppendorf AG, Barkhausenweg, 22339; Hamburg, Germany) at 9000 rpm for 10 min. After centrifugation, the supernatant was obtained and stored for further analysis.

#### 2.2.2. Determination of the Total Phenolic Compounds of Sunflower Cake

The TPC of the extract was determined using the Folin–Ciocalteu method [32]. Briefly, 200 μL of sunflower cake phenolic extract (SFPE) and 1.58 mL of pure water were added to 100 μL of Folin–Ciocalteu reagent (1:10, *v*/*v*). After 8 min at 25 °C in the dark, 300 μL of 20% sodium carbonate was added. The mixture was vortexed, then incubated at 40 °C for 30 min, and the absorbance at 765 nm was recorded on a UV–Vis spectrophotometer (SPECTRO star^®^ Nano microplate, Ortenberg, Germany). The TPC was expressed as mg gallic acid equivalent (GAE)/100 g dry matter of the sample, utilizing a gallic acid standard curve with a coefficient of determination (R^2^) of 0.99.

#### 2.2.3. DPPH Free Radical-Scavenging Activity of SFPE

The DPPH free radical scavenging activity of SFPE was evaluated according to the method described by Pandey et al. [33] with some modifications. In total, 50 μL of the samples at various concentrations were dissolved in methanol and then added to 2 mL of a 60 mM methanol solution of DPPH. The absorbance was recorded at 517 nm after 20 min at room temperature in the dark. Methanol with DPPH solution was used as a control, and the inhibition percentage of the DPPH free radical was calculated using:

$$\%\;\mathrm{DPPH}\;\mathrm{scavenging}\;=\;\frac{AControl-ASample}{AControl}\;\times 100$$

where Acontrol is the absorbance of the DPPH solution without the sample, and Asample is the absorbance in the presence of Kefiran. All measurements were carried out in triplicate.

### 2.3. Kefir Sample Preparation

Fresh kefir grains purchased from My Planet Store, Russia, were rinsed thoroughly with deionized water and inoculated into 2.5% ultra-pasteurized milk under aerobic conditions at room temperature for 72 h. After fermentation, the grains were separated from the fermented milk using a previously washed sieve rinsed with deionized water, and gently rinsed to remove residual milk.

#### Extraction and Isolation of Kefiran

**Figure 1:**
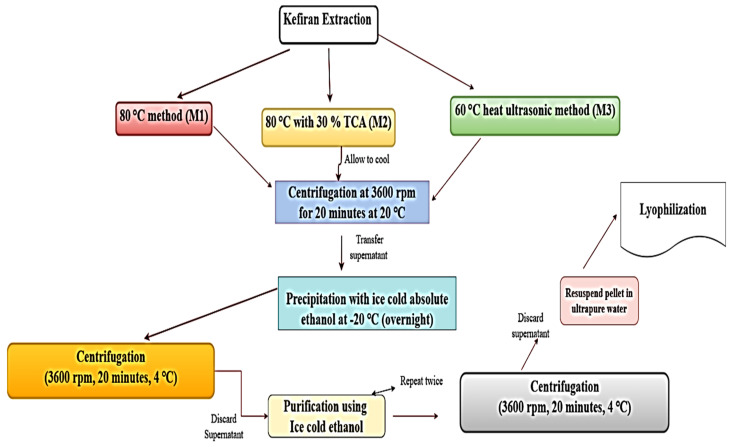
Schematic illustration of Kefiran extraction from the three different methods investigated.

Kefiran was extracted from kefir grains using the procedure described by La Torre et al. [34] with slight modifications, as summarized in Figure 1. Three extraction methods were applied using a grain-to-water ratio of 1:10 (*w*/*v*). In the hot-water method (M1), kefir grains were dispersed in distilled water and heated in a water bath (Termex, model LB62, Tomsk, Russia) at 80 °C for 10 min with intermittent shaking every 1 min. The other method included a hot-water treatment with 30% TCA (M2). Kefir grains were treated as in M1, and the dispersion was cooled to room temperature before 30% (*w*/*v*) trichloroacetic acid was added to promote protein denaturation and precipitation. The last method involved the mild heat ultrasonic method (M3), where kefir grains were suspended in distilled water preheated to 60 °C and sonicated for 10 min at 40 kHz and 120 W.

Following extraction, all samples were cooled to room temperature and centrifuged at 3600 rpm for 20 min at 20 °C to remove insoluble material. The supernatants were transferred into new tubes, followed by precipitation with ice-cold absolute ethanol at −20 °C (1:1 *v*/*v*) overnight. The precipitates were collected by centrifugation (3600 rpm, 20 min, 4 °C), and the supernatant was discarded. The pellet was resuspended in ice-cold absolute ethanol and centrifuged under the same conditions; this washing was repeated twice to obtain a white sediment. The sediment was air-dried to remove residual ethanol, redissolved in an appropriate volume of distilled water depending on pellet size, and finally lyophilized to obtain dry Kefiran powder.

### 2.4. Characterization of Kefiran

#### 2.4.1. Determination of Total Carbohydrates (Phenol–Sulfuric Assay)

The total carbohydrate content of the Kefiran samples was quantified by the phenol–sulfuric acid colorimetric method described by Dubois [34,35], using glucose as a calibration standard over the range of 20 to 120 µg/mL (y = 0.0016x − 0.0162, R^2^ = 0.9522). The polymer solution (10 µL, 1 mg/mL) was added to a phenol solution (500 µL, 5% *w*/*v*) and sulfuric acid (2 mL), and the mixture was heated in a water bath at 100 °C for 15 min. The reaction mixture was cooled to room temperature, and the absorbance was measured at 490 nm using a UV-Vis spectrophotometer (SPECTROstar^®^ Nano microplate, Ortenberg, Germany) in triplicate, with a blank containing 10 µL of water in place of the sample.

#### 2.4.2. Determination of Protein Content (Bradford Assay)

The total protein was determined using the Bradford colorimetric method [36], with bovine serum albumin as a standard, ranging from 20 to 120 µg/mL (y = 0.0025x + 0.2043, R^2^ = 0.9892). The polymer solution (10 µL, 1 mg/mL) was added to 100 µL of Coomassie Brilliant Blue reagent and incubated for 5 min at room temperature in the dark. The absorbance of the resultant blue color was measured at 595 nm using a UV-Vis spectrophotometer (SPECTROstar^®^ Nano microplate, Ortenberg, Germany). The measurement was conducted in triplicate using 10 µL of distilled water as the blank.

#### 2.4.3. Determination of Water-Holding Capacity (WHC)

The water-holding capacity of the samples was determined using a reported method [37], with modifications. Fine powdered Kefiran (0.1 g) was dispersed in 10 mL of distilled water and stirred for 2 h at 25 °C to obtain a uniform solution. The resulting solution was centrifuged at 13,000 rpm for 30 min, and the precipitate was collected. The water-holding capacity was calculated using the formula:

$$\%\;\mathrm{WHC}\;=\;\frac{Water\;bound\;weight}{Total\;dry\;sample\;weight}\;\times 100$$

#### 2.4.4. Fourier Transform Infrared (FTIR) Characterization

Fourier transform infrared spectroscopy analysis was carried out on the lyophilized extract to determine the vibrational modes of the various samples, recorded over 4000–400 cm^−1^ at 4 cm^−1^ intervals [37,38], using a Nicolet™ Is™ 5 spectrometer (Thermo Fisher Scientific, Waltham, MA, USA). Powdered samples of Kefiran were mixed with powdered potassium bromide in a ratio of 1:100 and molded into transparent pellets.

#### 2.4.5. Thermogravimetric Analysis (TGA)

Thermogravimetric analysis of the Kefiran samples was performed according to the method described by Saimaiti & Gençcelep [39], with minor modifications. The samples were analyzed using a thermogravimetric analyzer (NETZSCH TG 209F1 Libra (TGA), Selb, Germany) over a temperature range of 25–900 °C, with a continuous nitrogen gas flow of 50 mL/min and a heating rate of 10 °C/min.

#### 2.4.6. Morphological Characterization

The surface morphologies of the extracted polysaccharides were examined using scanning electron microscopy (SEM) (Quanta 200 method, FEI, Hillsboro, OR, USA). The imaging was performed using a wide-area low-vacuum secondary electron detector (LFD, Hillsboro, Oregon) at 2.00 kV and 70 Pa. The extracts were coated with gold for SEM. Images of morphology were obtained from the scattered electron (SE) signal.

### 2.5. DPPH Radical Scavenging Activity of Kefiran

The antioxidant activity of the extracted Kefiran exopolysaccharides was assessed by DPPH (2,2-Diphenyl-1-picrylhydrazyl) free radical scavenging activity described by Yusuf et al. [40]. A stock Kefiran solution was prepared by dissolving 0.1 g of lyophilized Kefiran in 10 mL of distilled water, and aliquots of different concentrations (0.2, 0.4, 0.6, 0.8, and 1.0 mg/mL) were obtained. Subsequently, 2 mL of the samples were mixed with 1 mL of DPPH ethanolic solution (0.1 mM). The mixtures were vortexed briefly and incubated in the dark at room temperature for 30 min, after which the absorbance was recorded at 517 nm. Vitamin C was used as a positive control. The percentage of DPPH scavenging was calculated using the formula:

$$\%\;\mathrm{DPPH}\;\mathrm{scavenging}\;=\;\frac{A\;Control-\;A\;Sample}{AControl}\;\times 100$$

where A_control_ is the absorbance of the DPPH solution without Kefiran, and A_sample_ is the absorbance in the presence of Kefiran. All measurements were carried out in triplicate.

### 2.6. Encapsulation Using Coacervation Complex

Complex coacervation encapsulation was conducted using the described method [40,41] with some modifications. Kefiran extract, gum arabic (GA), and whey protein isolate (WPI) were selected as the coating material to encapsulate phenolic compounds from sunflower cake extract. Three different formulations (Table 1) were generated, and the pH of each was adjusted to 3.75 by adding 0.1 M HCl dropwise. The formulations were made based on the ratio of Kefiran polysaccharide to whey protein isolate and GA to WPI. The procedure involved preparing each coating material solution at a concentration of 0.1 g/10 mL. The specific amounts of Kefiran polysaccharide, GA, WPI, and the phenolic compound used are reported in Table 1. The phenolic compound was dissolved in a 0.1 g/10 mL WPI solution using a magnetic stirrer at 500 rpm for 2 min. After complete dissolution, Kefiran polysaccharide was slowly added dropwise to the solution. The pH was adjusted to 3.75 to enhance the electrostatic attraction between Kefiran polysaccharide and whey protein isolate. This was repeated to form a coacervation complex between gum arabic and WPI. The resulting coacervate dispersions were allowed to equilibrate and subsequently stored under refrigerated conditions for further characterization.

### 2.7. Characterization of the Coacervate Complex

#### 2.7.1. Encapsulation Efficiency

The encapsulation efficiency was calculated by determining the phenolic content before and after encapsulation, as described by El-Messery [38]. The complex mixture was centrifuged at 3600 rpm for 15 min, and the supernatant containing the phenolic compound (nonencapsulated) was examined. The encapsulation efficiency (EE) was calculated using the following formula:

$$EE\;=\frac{\;TPC\;-\;FPC}{TPC}\times \;100$$

TPC: Total phenolic compounds (encapsulated + nonencapsulated).

FPC: Free phenolic compounds in the supernatant (nonencapsulated).

#### 2.7.2. Determination of Particle Size and Zeta Potential (ζ)

The particle size of the carrier particle was determined by diluting the capsule with distilled water, and the zeta potential was measured using a zetasizer instrument.

### 2.8. Antimicrobial Activity of Kefiran and Kefiran Coacervate Complex

#### 2.8.1. Preparation of Microbial Cultures

Test strains of microorganisms (*S. aureus* ATCC 6538-P, *E. coli* ATCC 25922, *P. aeruginosa* ATCC 9027, and *C. albicans* ATCC 10231) in ampoules obtained from official collections with certificates of conformity were used. The ampoules were opened under aseptic conditions according to the manufacturer’s instructions, and the cultures were restored to viability in a suitable medium, depending on the test microbial culture. Incubation was performed at optimal temperatures (35–37 °C for bacteria and 23–25 °C for fungi).

#### 2.8.2. Antimicrobial Activity Using the Agar Plate Dilution Method

The agar plate dilution method based, on the direct determination of MIC (minimum inhibitory concentration), was employed for the study. Different concentrations (250–1000 µg/mL) of Kefiran extract and its encapsulated form were used in this study. Briefly, 2 mL of each dilution was added to each test tube containing 2 mL of nutrient medium. After serial dilution, 1 mL of microbial inoculum (*S. aureus* ATCC 6538-P, *E. coli* ATCC 25922, *P. aeruginosa* ATCC 9027, and *C. albicans* ATCC 10231) was added to each test tube, along with 1 mL of the sample. A parallel control (without the sample) was set up. The test tubes were sealed with sterile stoppers and incubated in a thermostat, depending on the test microorganism.

### 2.9. Statistical Analysis

The experiments were performed in triplicate and reported as the mean ± standard deviation. Statistical analysis was performed using a one-way ANOVA test, followed by a Tukey post hoc test with *p* < 0.05.

## 3. Results and Discussion

Following the described methodology, phenolic compounds were successfully extracted from sunflower cake and subsequently encapsulated; the Kefiran extract was influenced by the extraction methods investigated. The physicochemical properties of the Kefiran extract were successfully determined by WHC, FTIR, TGA, and SEM analyses. Kefiran can be used as an encapsulating material to protect bioactive compounds and to inhibit the growth of certain harmful microorganisms.

### 3.1. Total Phenolic and Antioxidant Activity of Sunflower Cake Extract

The phenolic compounds in sunflower cake were successfully extracted using UAE and quantified using Gallic acid as a standard. The resulting concentration was expressed as GAE mg/L, with mean ± SD (Table 2). The results show that ultrasound-assisted extraction for 20 min exerts superior efficiency in extracting phenolic compounds, likely due to effective cell disruption by cavitation [41]. The antioxidant activity of the SFPE was investigated using DPPH (2,2-diphenyl-1-picrylhydrazyl) and expressed as the percentage inhibition of the DPPH radical (Table 2). The SFPE was found to have strong free radical scavenging activity, which is in line with reported studies [42], which showed that phenolic compounds from sunflower florets recorded high antioxidant activity of 1531.7 ± 203.2–1817.0 ± 51.5 using distilled water as a solvent.

### 3.2. Determination of Total Carbohydrate and Protein Content

Spectrophotometric analysis revealed notable differences in the carbohydrate and protein contents of Kefiran extracts prepared using different extraction methodologies (Table 3). The highest carbohydrate concentration was obtained with the ultrasonic water bath at 60 °C (M3), yielding 81.4 ± 0.9 mg/mL, followed by the water bath at 80 °C (M1) with 78.35 ± 1.63 mg/mL. The lowest yield was recorded for the water bath at 80 °C with prior treatment using 30% TCA (M2). Statistical analysis indicated significant differences between M1 and M2 and between M3 and M2, whereas no significant difference was observed between M1 and M3. These findings are consistent with previous reports by Pandey et al. [33], which demonstrate that ultrasonic-assisted extraction at mild temperatures promotes optimal Kefiran recovery, shortens processing time, and preserves the chemical integrity of the biopolymer. Lower yields in TCA-containing protocols are consistent with earlier studies [43], which reported that up to 50% of exopolysaccharides (EPSs) can co-precipitate during TCA treatment, resulting in considerable losses. Protein content followed a similar extraction-dependent pattern, with the highest concentration observed in M3 (5.69 ± 0.58 mg/mL), followed by M1 (4.45 ± 0.49 mg/mL) and M2 (2.23 ± 0.38 mg/mL). The predominance of carbohydrates over proteins in all extracts supports the polysaccharide-rich nature of Kefiran and the efficiency of the extraction methods used, as verified by spectrophotometric analysis.

### 3.3. Water-Holding Capacity (WHC)

Water-holding capacity describes the ability and behavior of polysaccharides to absorb and retain water per unit weight of dry polysaccharide, primarily due to hydrophilic functional groups and polymer network formation. The WHC values presented in this study suggest that the three Kefiran extracts have significantly different water absorption and retention mechanisms based on their varied microstructures. Values of WHC reported in published works on Kefiran gels and cryogels typically range from 70 to 95%, with more open, sponge-like structures that absorb water within pores [44]. The WHC is calculated to be 85% (M1), 77% (M2), and 71% (M3). The variations in WHC can be attributed to differences in extract porosity resulting from the different extraction conditions investigated. This result shows an improved water-holding capacity compared to exopolysaccharides extracted from beef sausage (8.95%) and from *Leuconostoc lactis* (7.5%) [45,46]. The highest WHC value recorded (M1) can be attributed to the heating condition, which maintained molecular weight and porosity, as observed in (M2) with a TCA inclusion treatment that partially hydrolyzed the polysaccharide. The synergy between ultrasound and heating intensifies shear and cavitation effects, resulting in significant depolymerization and structural fragmentation of the polysaccharides. This leads to a more open, interconnected pore structure that, although highly porous, favors water drainage rather than retention [47].

### 3.4. Fourier Transform Infrared Spectroscopy Characterization

**Figure 2:**
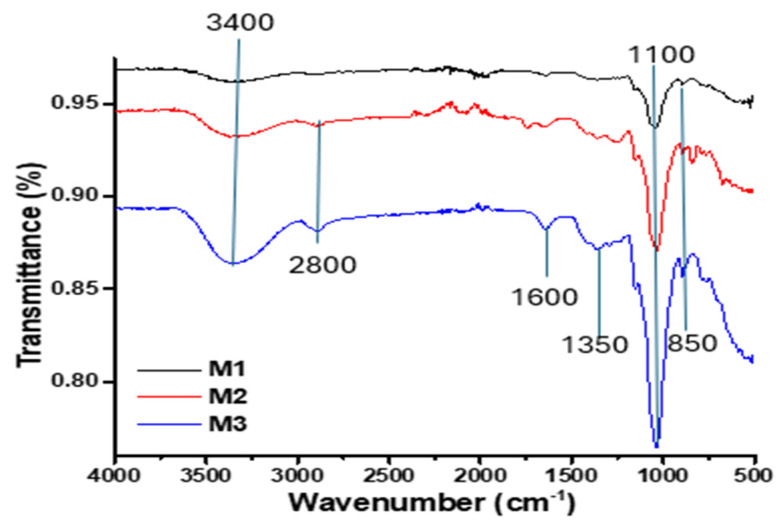
Fourier transform infrared spectroscopy of Kefiran (M1: hot water bath at 80 °C, M2: hot water bath at 80 °C with 30% TCA precipitation, and M3: ultrasonic water bath at 60 °C).

FT-IR spectra of Kefiran polysaccharides extracted using three methods (M1: hot water bath at 80 °C, M2: hot water bath at 80 °C with 30% TCA precipitation, and M3: ultrasonic water bath at 60 °C) revealed characteristic absorption peaks across the 3400 to 840 cm^−1^ range (Figure 2), consistent with previously reported Kefiran profiles.

A prominent, broad absorption peak observed around 3400 cm^−1^ corresponds to hydroxyl (O–H) stretching vibrations associated with bound water and polysaccharide hydroxyl groups. The ultrasonic-mild heat extraction method (M3) exhibited the highest intensity at this peak, indicating better preservation or higher content of hydroxyl-rich polysaccharide structures, corroborating findings by Shen et al. [48] and Wang et al. [49], who reported enhanced extraction efficiency and polysaccharide integrity using ultrasound-assisted mild heating. The weak peak around 2800 cm^−1^ corresponds to C–H stretching vibrations, which are typical polysaccharide markers and are observed consistently across all methods [49]. Absorbance in the 1600 cm^−1^ region, associated with amide I and II bands arising from C=O stretching and C–N bending of proteins and peptides, was notably reduced in M1 and M2 extracts. This decrease is attributed to protein removal by TCA precipitation in M2 and possible thermal denaturation effects in M1, as similarly reported by Li et al. [50]. These results confirm that TCA effectively reduces protein content but may alter polysaccharide–protein interactions. The absorption peak observed around 1350 cm^−1^ was assigned to C-H deformation vibrations. The absorption peak around 1100 cm^−1^ corresponds to C-O-H stretching vibrations, a fingerprint region of polysaccharides [51,52]. The absorption peak in the 850 cm^−1^ region is a representation of the α- and β-type glycosidic bonds.

### 3.5. Thermogravimetric Analysis (TGA)

**Figure 3:**
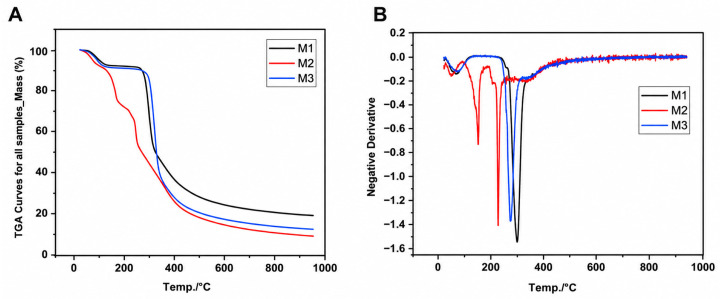
(**A**) Thermogravimetric analysis (TGA) of Kefiran exopolysaccharides (M1, M2, M3). (**B**) Differential thermogravimetric analysis (DTG) of Kefiran exopolysaccharides (M1, M2, M3).

Thermogravimetric analysis (TGA) and its first derivative (DTG) were used to study the thermal stability and decomposition behavior of the Kefiran extract under a controlled atmosphere. This technique measures changes in weight as the sample is heated, revealing its characteristics based on the polysaccharide’s composition and structure. The thermogravimetric (TG) and differential thermogravimetric (DTG) curves of Kefiran exopolysaccharides, depicted by the percentage change in mass with increasing temperature, are presented in Figure 3, along with the onset and maximum degradation temperatures (T_onset_ and T_max_ in °C; Table 4). The TGA curves from all extracts (M1, M2, and M3) show multiple degradation steps, a feature commonly observed in polymers. During the initial heating stage from room temperature to 200 °C, the TGA curves for all extracts showed a gradual, slight mass loss, corresponding to the release of low-molecular-weight compounds and residual solvents. Kefiran exopolysaccharides from M1 and M3 exhibited two degradation steps, while the Kefiran exopolysaccharide from M2 exhibited three degradation steps. The method employed in M3 shows the highest thermal stability, with maximum degradation at 305 °C and an initial onset at 70 °C, confirming its ability to withstand the highest temperatures before significant structural breakdown. The multistep decomposition behavior of M3 can be linked to the presence of a highly stable polymer matrix. Kefiran exopolysaccharide from M1 shows close correspondence, with a maximum degradation peak at 277 °C and an initial degradation at 76 °C. While its onset was similar to that of M3 in the TGA, the DTG clearly shows that M1 reaches its maximum degradation rate and undergoes rapid degradation, often due to backbone breakdown. Sample M2 shows a complex, multistep degradation with more labile components, which differs from most DTG reports on Kefiran exopolysaccharides. The presence of two major peaks at 155 °C and 230 °C suggests a sequential loss of distinct components, with an initial degradation at 60 °C. The major thermal decomposition observed around 230–305 °C is mostly attributed to the Kefiran biopolymer, consistent with reported studies [53,54]. The onset degradation temperatures of all three extracts for M1 (76 °C), M2 (60 °C), and M3 (70 °C) are lower than those reported for some other microbial exopolysaccharides [53]. Still, this early onset is mainly due to the loss of loosely bound structural water rather than polymer backbone degradation [54], as is typical of hydrophilic polysaccharides with a high density of hydroxyl groups. The most analytically meaningful parameter was Tmax, the temperature at which the decomposition rate was maximal; M3 had the highest Tmax of 305 °C, compared to 277 °C for M1 and 230 °C for M2. This confirms that ultrasound-assisted mild heat extraction produces a Kefiran matrix with superior thermal stability of the polysaccharide backbone, most likely due to improved preservation of high-molecular-weight polymer chains and a more ordered network structure, as indicated by the FTIR hydroxyl band intensity.

The total mass percentage of the exopolysaccharides (Table 4) is consistent with most polysaccharides [55,56].

### 3.6. Scanning Electron Microscopy

The surface morphology of the extracted Kefiran polysaccharide was examined by scanning electron microscopy to reveal its distinct physical characteristics. All the samples extracted using different methods exhibited some level of porosity, an indicative characteristic of the water-holding capacity of polysaccharides [57]. SEM images and WHC measurements revealed a clear inverse correlation between the surface porosity and water retention of the three extracts. M3 has the lowest WHC of 71%, but it has the most open and linked porous network. M1, with an uneven spongy structure and less connected pores, retained more water with a WHC of 85%. Differences in open and closed porosity may explain this pattern. The open-pore architecture of M3 is highly interconnected, favoring drainage over water retention. The closed pores of M1 are irregularly shaped and physically retain water inside the matrix. The results are analogous to those in papers on other biopolymer systems, where water-holding behavior is governed by pore connectivity rather than porosity alone.

**Figure 4:**
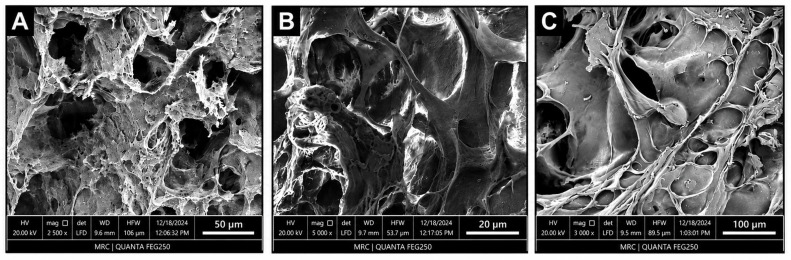
Surface morphology image of extracted Kefiran captured by scanning electron microscopy. (**A**) M1; (**B**) M2; (**C**) M3.

Aside from their porosity, the M1 extract exhibited a rough, spongy-like structure with irregular voids and lower compactness (Figure 4A). M2 samples or extracts exhibited a smooth surface and a less compact structure, with some level of densification (Figure 4B). Extracts from M3 had a smooth surface with less dense, interconnected thin walls (Figure 4C). These similar morphological characteristics were observed in exopolysaccharides produced from thermophilus spp., *B. anthracis*, and a mesophilic spp., *B. licheniformis* [58,59]. Differences in extraction and processing methods directly modify surface compactness, porosity, and wall connectivity, as seen in the Kefiran extract. The alteration in microstructure is linked to the extraction temperature and duration, which likely modified the polymer network due to extended thermal treatment, resulting in the observed morphological differences.

Thus, the morphological differences observed between the three Kefiran extraction methods—rough spongy (M1), smooth densified (M2), and smooth interconnected porous (M3)—correspond to a report by Matos et al. [60]. These surface features have significant implications for functional properties, such as water-holding capacity, mechanical strength, and suitability for tissue engineering and food packaging applications, as well as strong biofilm-forming abilities.

### 3.7. DPPH Radical Scavenging Activity

**Figure 5:**
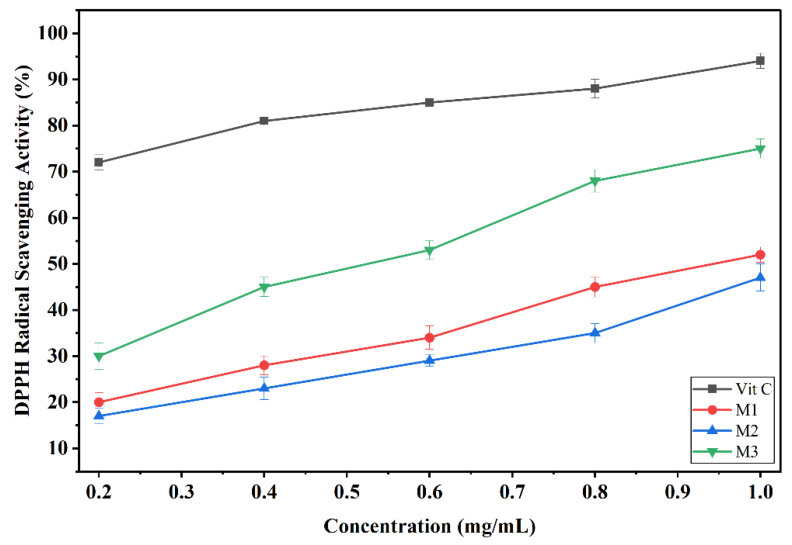
DPPH radical scavenging activity of Kefiran exopolysaccharide influenced by different concentrations and different extraction methods.

Kefiran exhibited a clear concentration-dependent DPPH radical scavenging profile, with inhibition increasing from 17% (M2, 0.2 mg/mL) to 75% (M3, 1.0 mg/mL) (Figure 5). This pattern mirrors similar observations for other kefir-derived exopolysaccharides, where radical scavenging increases as the concentration of Kefiran increases. The results show that the exopolysaccharide produced by M3 (ultrasound-assisted extraction) recorded the highest percent scavenging activity (30–75%), while the exopolysaccharide produced by M2 (TCA + heat treatment) recorded the least scavenging activity (17–44%) with increasing dose concentration. The exopolysaccharide from M1 (heat-treated method) showed antioxidant activities of 20–46% at increasing dose concentrations, which were higher than those observed with the M2 method. In addition, these results showed that the inhibitory activity of Kefiran exopolysaccharide was lower than that of vitamin C (positive control) at all tested concentrations. Although these percentage inhibition values are lower, on a mass basis, than those usually reported for small-molecule antioxidants, they confirm that ultrasound-extracted Kefiran retains appreciable radical-quenching capacity. The IC_50_ value for the control (vitamin C) was <0.2 mg/mL, indicating strong antioxidant activity. Sample M3 recorded an IC_50_ value of 0.523 mg/mL, while M1 and M2 recorded an IC_50_ value of >1.0 mg/mL. These results indicate that M3 requires a lower concentration to achieve 50% radical inhibition than M1 and M2, which require higher concentrations. The antioxidant activity of exopolysaccharides has been attributed to their structural and functional properties, including the amounts of proteins, carbohydrates, amino acids, carbonyl, and carboxylic groups [61,62], with hydrogen-donating ability influencing their activity against free radicals. This result is consistent with that of Oliveira Filho et al. [13], who reported that kefir exopolysaccharide, when treated with ultrasound followed by heat, showed the highest DPPH antioxidant scavenging activity. Within the framework of the present work, this concentration-dependent DPPH activity further underscores the role of Kefiran as a multifunctional component of the coacervate wall material, contributing to both the physical integrity of the capsules and additional antioxidant protection for encapsulated sunflower-cake phenolic compounds in prospective functional food applications.

### 3.8. Characterization of Capsules

#### 3.8.1. Particle Size and Zeta Potential ζ

Measurements of particle size and zeta potential (Table 5) were performed for the different formulations created by combining WPI with various polysaccharides designated G, M1, M2, and M3 at varying weight ratios (1:0, 1:1, and 1:2). These evaluations are essential when assessing the physical properties and stability of encapsulated systems, which are frequently utilized in food and medicinal applications [63]. Particle sizes and zeta potentials were recorded for the formulations studied during coacervation complex formation. This can be attributed to the different materials studied, the extraction methods, and the ratio of polysaccharides to WPI during wall material formation. Formulations in G: WPI resulted in fewer particles as WPI concentration increased, which, in turn, affected the charge of the encapsulate formed. The formulation G: WPI (1:2) resulted in a smaller size of 71.90 ± 10.39 nm, with a zeta potential of −33.85 mV, indicating less aggregation and good stability of the capsules formed from this formulation. Similar observations were made for M2 and M3 samples; increasing the WPI concentration decreased particle size, but M2 showed greater consistency in size distribution than M3. The highest particle size was observed for M3: WPI (1:0), at 2.2 × 10^4^ ± 1.1 × 10^4^ nm. G suggests better dispersion or stabilization at higher WPI content: WPI (1:2). Higher WPI ratios often result in particle size reduction and stability in G, M1, M2, and M3 samples (1:2). Particle size and surface charge differences between M1, M2, and M3 suggest variations in their formulations and treatments. The ζ-potential values differed across formulations and were mostly negative under the acid coacervation conditions at pH 3.75. However, the WPI ratios of 1:1 and 1:2 in M1-based formulations exhibited near-zero or slightly positive zeta potentials (+0.90 and +3.07 mV, respectively), indicating that charge neutralization or overcompensation occurred at higher WPI concentrations. This is consistent with studies indicating that an increase in protein content in the polysaccharide–protein coacervate systems shifts the surface charge toward neutrality or positive values when the positive charge of WPI at an acidic pH dominates the complex interface. Formulations with near-zero zeta potential exhibit reduced electrostatic repulsion between particles, potentially enhancing aggregation and diminishing colloidal stability, which could explain the relatively larger particle sizes observed in M1-based formulations.

The physical stability of encapsulates is indicated by the negative ζ-potential values, which imply repulsive forces between the particles that can prevent aggregation [40,64]. On the other hand, complex particles may aggregate and form complex coacervates if their ζ-potential is reduced to near zero.

#### 3.8.2. Encapsulation Efficiency

**Figure 6:**
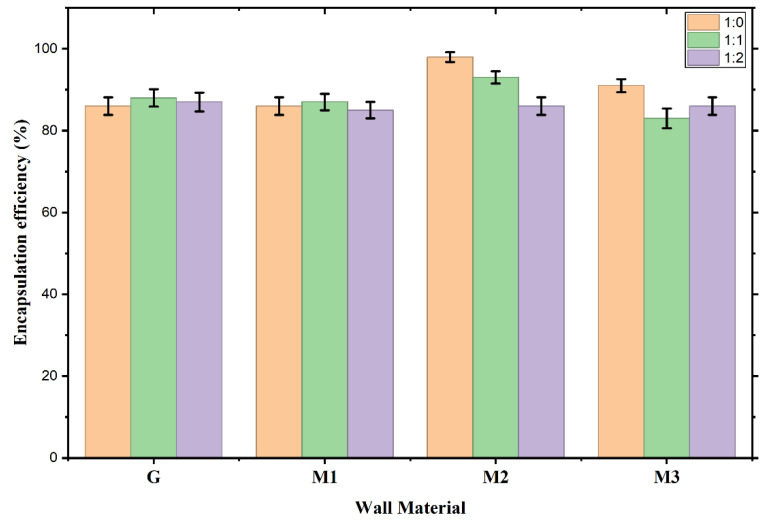
Encapsulation efficiency of extracted Kefiran polysaccharide (M1, M2, M3) vs. gum arabic (G) in their different proportions.

The encapsulation efficiency describes how effectively the different polysaccharides combined with WPI influence the encapsulation process. For core material to be well encapsulated, a high EE (%) is essential. Complex coacervates with lower surface phenolic content exhibit more effective encapsulation. All samples exhibited high encapsulation efficiency, ranging from 83% to 93% (Figure 6). Increasing the WPI ratio to (1:1) or (1:2) generally maintains high efficiency; however, there is a tiny loss in some circumstances (particularly for M3:WPI). Interestingly, in most formulations, the polysaccharide alone at a 1:0 ratio achieved the highest or near-maximum encapsulation efficiency without WPI. This does not diminish the role of WPI in the coacervate system, but suggests that at high ratios of WPI, competitive adsorption and partial saturation of polysaccharide binding sites may slightly decrease phenolic retention at the wall material interface. In these formulations, WPI primarily promotes electrostatic complexation and coacervate formation, thereby contributing to colloidal stability, particle size control, and mechanical shell integrity. Therefore, the marginal reduction in encapsulation efficiency at higher WPI ratios should be regarded in the context of the overall improvement in coacervate stability and structural performance rather than as evidence against WPI inclusion.

### 3.9. Antimicrobial Activity

#### 3.9.1. Antimicrobial Activity of Kefiran Extract

The antimicrobial activity of the extracted Kefiran polysaccharide was investigated using the agar plate dilution method to determine the minimum inhibitory concentrations at which the extract inhibits the growth of *S. aureus, E. coli, P. aeruginosa*, and *C. albicans*. The MIC ranged from 250 µg/mL to 1000 µg/mL against the tested microorganisms (Table 6). This suggests that Kefiran can be used as an antimicrobial agent to treat *S. aureus*, *E. coli*, *P. aeruginosa*, and *C. albicans* [65]. The antimicrobial activity in this study shows a distinct spectrum of inhibition that varies with the target microorganism. Based on the MIC results, sample M3 demonstrated the strongest inhibitory effect against *S. aureus* ATCC 6538-P at 250 µg/mL, followed by M1 (500 µg/mL), with M2 showing the least inhibitory effect at 1000 µg/mL. This range is notably lower than the previous MIC of 5000 µg/mL reported for exopolysaccharide extracted from water kefir against *S. aureus* [66]. Ultrasound extraction with mild heat (M3) recorded the minimum inhibitory concentration (250 µg/mL) against *S. aureus*, indicating that this extraction method better preserves bio-functional activities. However, the MICs of the extracts against *P. aeruginosa* ATCC 9027 ranged from 500 µg/mL (M1, M3) to 1000 µg/mL (M2), reflecting moderate antibacterial activity due to its impermeable outer membrane and efflux system. Similarly, *E. coli* ATCC 25922 showed MIC values from 500–1000 µg/mL, with M2 demonstrating stronger inhibition at 500 µg/mL. The relatively higher MIC for this Gram-negative bacterium can be attributed to its complex cell membrane structure and lipopolysaccharide-rich outer membrane, which reduces cell permeability [67]. M2 exhibited a greater inhibitory effect against *E. coli* (MIC 500 µg/mL) than M3 (MIC 1000 µg/mL). M2 has a unique activity against *C. albicans* (MIC 500 µg/mL) that was not detected in M1 and M3. The differences may be due to chemical modifications induced by TCA treatment. The precipitation with TCA partially hydrolyzes the polysaccharide chain, which may expose or create functional groups, such as free carboxyl and hydroxyl groups, that are more compatible with the lipopolysaccharide-rich outer membrane of *E. coli* and the β-glucan-containing cell wall of *C. albicans*. This suggests that ultrasound-assisted extraction (M3) is overall the better approach for preserving bioactive integrity and broad antibacterial performance, while the TCA-induced structural modification in M2 might provide an alternative pathway for improving activity.

Overall, the results demonstrate the ability of Kefiran polysaccharide to exhibit potent and selective antimicrobial effects, consistent with the study by Al-Mohammadi et al. [67].

#### 3.9.2. Antimicrobial Activity Using Agar Plate Dilution Method of Coacervates

The agar plate dilution method using the minimum inhibitory concentration (MIC) was employed because it provides a clear visual assessment of whether coacervate complexes can inhibit the growth of specific microorganisms at specific concentrations. This assay establishes a quantitative threshold (MIC value) to perform its functional activities. It is a widely accepted method for evaluating biopolymer-based antimicrobials and is suitable for thick or granular systems, such as Kefiran-WPI coacervates. By applying test samples at precise doses and observing their diffusion into the agar, a local gradient forms, indicating both the presence and the strength of inhibition. The MICs of the coacervates showed that none of the formulations were effective against *P. aeruginosa* ATCC 9027 and *C. albicans* ATCC 10231 (Table 7). Coacervate complex from G: WPI formulations inhibited the growth of *E. coli* ATCC 25922 at an MIC value of 1000 µg/mL and *S. aureus* at 500–1000 µg/mL, while M3:WPI achieved an MIC below 1000 µg/mL against *E. coli* and *S. aureus* ATCC 6538-P. This suggests that G and M3 preparations form coacervates with more favorable microstructures for antimicrobial action. In contrast, M2: WPI complexes showed MICs against *E. coli* of less than 1000 µg/mL. This suggests that variations in Kefiran origin, purification, or molecular conformation among G, M1, M2, and M3 significantly impact the functional characteristics of the resulting coacervates.

The antimicrobial activity of Kefiran–WPI coacervates is governed not only by their chemical composition but also by their microstructure, charge distribution, and diffusion ability through the agar matrix. Protein–polysaccharide coacervates formed by the interaction of proteins and polysaccharides typically exert antimicrobial effects through a combination of electrostatic interactions with microbial cell surfaces, membrane permeability, and localized concentration gradients at the site of contact, rather than through classical diffusion-based antibiotic mechanisms.

The lack of inhibitory activity against *P. aeruginosa* ATCC 9027 and *C. albicans* ATCC 10231 can be attributed to their intrinsic resistance mechanisms conferred by their cell wall components and protective capabilities. *P. aeruginosa* possesses a highly impermeable outer membrane, efficient efflux pumps, and strong biofilm-forming capacity, which collectively limit the penetration and effectiveness of macromolecular antimicrobial systems [68,69]. Similarly, *C. albicans* exhibits a rigid, polysaccharide-rich cell wall and adaptive stress responses that reduce susceptibility to polymer-based antimicrobials [70].

In contrast, coacervates formed from G:WPI and M3:WPI formulations demonstrated inhibitory activity against *E. coli* and *S. aureus*, which can be attributed to the antimicrobial properties conferred by the system. Specifically, Kefiran–WPI coacervates with optimal charge balance and less compact microstructures may enhance interaction with bacterial cell envelopes, promoting membrane destabilization or nutrient restriction at the agar–cell interface. Gram-positive bacteria, such as *S. aureus*, are particularly susceptible to these mechanisms because they lack an outer membrane. At the same time, Gram-negative *E. coli* remains moderately sensitive when coacervate diffusion and surface interactions are sufficient [71].

The limited activity of M2:WPI complexes, which inhibited only *E. coli*, suggests that differences in Kefiran molecular weight and chain branching may result in coacervates with less interactive networks. The diffusion properties of these compact structures through the gel matrix and their surface interactions with bacterial cells could be limited, resulting in diminished antimicrobial efficacy. Similar structure-activity relationships have been reported for polysaccharide–protein complexes, in which excessive compactness or reduced surface charge accessibility compromises antimicrobial performance.

Overall, these findings indicate that various Kefiran extraction methods can critically influence coacervate architecture, which, in turn, governs antimicrobial performance. Coacervates derived from G and M3 preparations appear to achieve a structural balance that favors microbial interaction and inhibition, whereas M2-derived systems may be less optimal due to unfavorable molecular conformation or network density.

## 4. Conclusions

This study provides a systematic comparison of hot water (M1), TCA-assisted hot water (M2), and ultrasound-assisted mild heat (M3) extraction protocols for Kefiran exopolysaccharide, directly relating extraction conditions to structural integrity, bioactive preservation, and functional performance in a phenolic delivery system, an integration not previously reported for Kefiran. FTIR, SEM, TGA, and WHC analyses confirmed the polysaccharide nature of all extracts while showing that extraction conditions critically determine functional group integrity, porosity, thermal stability, and water retention. The ultrasound-assisted extraction at 60 °C (M3) gave the highest carbohydrate yield. Its superior bioactive performance, with an IC_50_ of 0.523 mg/mL for DPPH scavenging and a MIC of 250 µg/mL against *S. aureus*, can be explained by acoustic cavitation effects that enhance cell wall disruption and polysaccharide release while avoiding the thermal degradation and co-precipitation losses associated with M1 and M2, respectively. These results are compared to previously reported MICs of 5000 µg/mL for water kefir EPSS against *S. aureus*, highlighting the functional benefit of ultrasound-assisted processing.

Sunflower cake phenolic compounds, a valorization opportunity for an abundant agricultural by-product, were successfully encapsulated in complex coacervates based on Kefiran at encapsulation efficiencies of 83–93%, comparable or superior to the values reported in the literature for whey protein–gum arabic systems (typically 75–90%) and confirming the competitiveness of Kefiran as a wall material. The selective antibacterial activity of Kefiran–WPI coacervates against *S. aureus* and *E. coli*, and their limited efficacy against *P. aeruginosa* and *C. albicans*, is indicative of the well-established resistance conferred by the outer membrane impermeability and efflux systems of Gram-negative and fungal organisms rather than a deficiency of the delivery system. These results suggest that the antibacterial activity of polymer-based systems is mostly determined by coacervate microstructure and surface charge distribution, which are controlled by the Kefiran extraction process.

Future research should focus on in vitro gastrointestinal digestion modeling and controlled-release profiling to evaluate the bioaccessibility of encapsulated sunflower cake phenolics under physiological conditions, along with systematic pH optimization of coacervate formation to improve encapsulation efficiency and colloidal stability. For a thorough quantification of the contribution of ultrasound energy to Kefiran bioactivity, a non-thermal, non-sonicated baseline extraction control will be required. Furthermore, the practicality and stability of these functional foods under food-relevant processing conditions (heat, light, storage) should be evaluated before converting these discoveries into functional foods or biomedical applications. Collectively, this work establishes the potential of ultrasound-extracted Kefiran as a high-performance, sustainably produced, multifunctional biopolymer and provides a replicable framework for integrating green extraction, structural characterization, and biofunctional validation in the design of next-generation bioactive delivery systems.

## Figures and Tables

**Table 1:** Wall material ratios used in complex coacervation formulations.

Formulation Set	Phenolic Compound (g)	Polysaccharide:WPI Ratio
GA:WPI	0.1	1:0; 1:1; 1:2
M1:WPI	0.1	1:0; 1:1; 1:2
M2:WPI	0.1	1:0; 1:1; 1:2
M3:WPI	0.1	1:0; 1:1; 1:2

**Table 2:** Total phenolic and antioxidant activity of sunflower cake extract.

Total Phenolic (GAE mg/L)	Antioxidant Activity (%)
726.5 ± 0.19	87.52 ± 0.04

Values are expressed as mean ± standard deviation of three replicates.

**Table 3:** The total concentration of carbohydrates and proteins.

Sample	Total Carbohydrate (µg mL^−1^)	Total Protein (µg mL^−1^)
M1	78.35 ± 1.63 ^A^	4.45 ± 0.49 ^a^
M2	55.07 ± 4.29 ^B^	2.23 ± 0.38 ^b^
M3	81.40 ± 0.90 ^A^	5.69 ± 0.58 ^a^

Values are expressed as means ± SD of three replicates. Different superscript letters within the same column indicate significant differences (*p* < 0.05). For total carbohydrate, (A, B) are used; for total protein, lower letters (a, b) are used.

**Table 4:** Thermal properties of Kefiran exopolysaccharides extracted using different methods (T_onset,_ onset temperature; T_max_, maximum temperature).

Sample	T_onset_ (°C)	T_max_ (°C)	Total Mass Loss (%)
M1	76	277	75
M2	60	155	78
		230	
M3	70	305	72

**Table 5:** Particle size of the encapsulate from the coacervation complex.

Material	Particle Size (nm)	Zeta Potential ζ (mV)
G: WPI (1:0)	374.0 ± 63.5	−19.73
G: WPI (1:1)	108.9 ± 40.62	−11.06
G: WPI (1:2)	71.90 ± 10.39	−33.85
M1: WPI (1:0)	386.0 ± 89.77	−5.28
M1: WPI (1:1)	713 ± 83.5	0.90
M1: WPI (1:2)	237.3 ± 42.65	3.07
M2: WPI (1:0)	507.5 ± 67.84	−4.12
M2: WPI (1:1)	152.7 ± 47.49	−4.43
M2: WPI (1:2)	139.4 ± 44.72	−2.01
M3: WPI (1:0)	281.8 ± 59.07	−1.62
M3: WPI (1:1)	241.8 ± 56.44	−4.55
M3: WPI (1:2)	158.7 ± 44.71	−1.05

Values are mean ± standard deviation of 3 replicates.

**Table 6:** Antimicrobial minimum inhibitory concentration of Kefiran.

Sample Name	*P. aeruginosa* ATCC 9027 (cc)	*E. coli* ATCC 25922 (µg/mL)	*S. aureus* ATCC 6538-P (µg/mL)	*C. albicans* ATCC 10231 (µg/mL)
M1	500	1000	500	Not active
M2	1000	500	1000	500
M3	500	1000	250	Not active

**Table 7:** Antimicrobial minimum inhibitory concentration of coacervates.

Sample Name	*P. aeruginosa* ATCC 9027 (µg/mL)	*E. coli* ATCC 25922 (µg/mL)	*S. aureus* ATCC 6538-P (µg/mL)	*C. albicans* ATCC 10231 (µg/mL)
G: WPI (1:0)	Not active	1000	<1000	Not active
G: WPI (1:1)	Not active	<1000	500	Not active
G: WPI (1:2)	Not active	<1000	1000	Not active
M1: WPI (1:0)	Not active	Not active	Not active	Not active
M1: WPI (1:1)	Not active	Not active	Not active	Not active
M1: WPI (1:2)	Not active	Not active	Not active	Not active
M2: WPI (1:0)	Not active	<1000	Not active	Not active
M2: WPI (1:1)	Not active	<1000	Not active	Not active
M2: WPI (1:2)	Not active	<1000	Not active	Not active
M3: WPI (1:0)	Not active	<1000	<1000	Not active
M3: WPI (1:1)	Not active	<1000	<1000	Not active
M3: WPI (1:2)	Not active	<1000	<1000	Not active

## Data Availability

The original contributions presented in this study are included in the article. Further inquiries can be directed to the corresponding authors.
